# Social and endocrine correlates of immune function in meerkats: implications for the immunocompetence handicap hypothesis

**DOI:** 10.1098/rsos.180435

**Published:** 2018-08-01

**Authors:** Kendra N. Smyth, Nicholas M. Caruso, Charli S. Davies, Tim H. Clutton-Brock, Christine M. Drea

**Affiliations:** 1 University Program in Ecology, Duke University, Durham, NC, USA; 2 Department of Evolutionary Anthropology, Duke University, Durham, NC, USA; 3 Department of Biology, Duke University, Durham, NC, USA; 4 Kalahari Research Trust, Kuruman River Reserve, Van Zylsrus, Northern Cape, South Africa; 5 Department of Biological Sciences, University of Alabama, Tuscaloosa, AL, USA; 6 Department of Zoology, University of Cambridge, Cambridge, UK; 7 Mammal Research Institute, University of Pretoria, Pretoria, South Africa

**Keywords:** androstenedione, ecoimmunology, immunocompetence handicap hypothesis, sexual selection, social dominance, testosterone

## Abstract

Social status can mediate effects on the immune system, with profound consequences for individual health; nevertheless, most investigators of status-related disparities in free-ranging animals have used faecal parasite burdens to proxy immune function in the males of male-dominant species. We instead use direct measures of innate immune function (complement and natural antibodies) to examine status-related immunocompetence in both sexes of a female-dominant species. The meerkat is a unique model for such a study because it is a cooperatively breeding species in which status-related differences are extreme, evident in reproductive skew, morphology, behaviour, communication and physiology, including that dominant females naturally express the greatest total androgen (androstenedione plus testosterone) concentrations. We found that, relative to subordinates, dominant animals had reduced serum bacteria-killing abilities; also, relative to subordinate females, dominant females had reduced haemolytic complement activities. Irrespective of an individual's sex or social status, androstenedione concentrations (but not body condition, age or reproductive activity) negatively predicted concurrent immunocompetence. Thus, dominant meerkats of both sexes are immunocompromised. Moreover, in female meerkats, androstenedione perhaps acting directly or via local conversion, may exert a double-edged effect of promoting dominance and reproductive success at the cost of increased parasitism and reduced immune function. Given the prominent signalling of dominance in female meerkats, these findings may relate to the immunocompetence handicap hypothesis (ICHH); however, our data would suggest that the endocrine mechanism underlying the ICHH need not be mediated solely by testosterone and might explain trade-offs in females, as well as in males.

## 1. Introduction

In humans, socioeconomic status (SES), determined by relative income, education and occupational position in society, is a strong predictor of disease incidence and mortality rates, such that each step down the SES ladder increases morbidity and decreases lifespan [1]. Status-related health differences, which may derive from status-related variation in immune function, are also prevalent in non-human animals: Membership in social groups is often stratified by dominance hierarchies and, like SES in humans, one's social standing can dramatically influence susceptibility to disease [1,2]. Nevertheless, the direction of the relationship between social status and immune function varies across species and, in some cases, across social contexts within a population [3–5]. In high-ranking individuals, the energetic cost of achieving and maintaining dominance, coupled with that of increased reproductive effort, may limit investment in immune defences [6–8]. By contrast, immunosuppressive correlates of low social status may include inadequate access to resources [9], lack of positive social interactions [10] and exposure to chronic social stress [11,12]. Because relationships between social status and immune function are complex and dynamic, predicting the direction of these relationships requires an understanding of the underlying biological mechanisms.

Among the mechanisms that have been invoked to explain status-related differences in immune function, endocrine mediators (e.g. glucocorticoids, or GCs, and androgens) have received considerable support [11–14]. Documented in males of many species [15–17], the trade-off between dominance (or reproductive success) and immunocompetence may be mediated by androgens, particularly testosterone (T) [13,14]. Indeed, T may affect the immune system directly, via binding to receptors on lymphocytes [18], or indirectly, via associated changes in other immunomodulatory hormones (e.g. GCs) [19], in energy allocation [20] or in behaviour [15,21]. Thus, T's antagonistic, pleiotropic effects (e.g. enhancing reproduction, but suppressing immune function) may create a health cost of male social dominance. To expand this area of study, we recently examined gastrointestinal parasitism in the cooperatively breeding meerkat (*Suricata suricatta*), a female-dominant species in which females are naturally, hormonally ‘masculinized’ [22], meaning that females, depending on their social or reproductive status, have either greater or equivalent androgen concentrations relative to males. We found that parasite load, an indirect measure of immune function, is positively linked to social status [23] and, in females, to concentrations of total androgens (i.e. combined androstenedione, or A_4_, and T, inferred from faecal androgen metabolites) [24]. Here, we build upon this work to test if social status or its various correlates, including serum concentrations of reproductive hormones, are related to two direct measures of innate immune function.

The innate immune system represents the first line of defence against pathogens; the ability to mount an appropriate immune response is an important aspect of disease resistance and, thus, survival. Two constitutively present components of innate immunity are complement and natural antibodies (NAbs). Complement is a group of serum proteins that opsonizes bacteria (making them susceptible to phagocytosis), initiates inflammation, and directly lyses foreign cells [25]. NAbs, in the presence of complement, are capable of lysis [26], but they can also neutralize some viruses and bacteria and stimulate adaptive immunity by enhancing antigen presentation to T and B cells [25]. Assays quantifying complement and NAbs are widely used by ecoimmunologists because they do not require species-specific reagents and, thus, are adaptable to non-model organisms [27–29]. Variation in complement and NAbs has been found to be positively associated with social status in spotted hyaenas, *Crocuta crocuta* [9], and rhesus macaques, *Macaca mulatta* [30].

We used meerkats as a model for investigating social correlates of immune function for two reasons. First, social status in this species is well advertised and we understand many of its morphological, behavioural, communicatory and physiological correlates under natural conditions [31–37]. Second, dominance is positively linked to reproductive success, which is highly skewed in both sexes [22,32,38,39]. Meerkats live in social groups or clans comprising a dominant, breeding pair and up to 40 adult and juvenile, subordinate helpers of both sexes. Social status is tightly linked to an individual's age [32] and weight [32,39]: When a dominance vacancy arises, intense competition ensues and the more senior individuals are more likely to be victorious over junior competitors [32,39,40]. Among females, but not males, heavier individuals gain an additional advantage in intrasexual dominance contests, perhaps indicating stronger selection for competitive abilities in females than in males [32,39,40]. Again, among females, but not males, dominance is associated with greater concentrations of sex hormones (i.e. A_4_, T and oestradiol or E_2_) [22,31–33]. A dominant female's raised hormone concentrations may be linked to increased aggressiveness, enabling her to control reproduction by thwarting potential rivals, forcibly evicting subordinate dams or killing their offspring [32,40,41]. Yet, the dominant pair relies on subordinate helpers to babysit, guard and provision pups [42], which entails significant foraging costs for subordinates, reflected in weight loss and reduced growth rates [42–44]. To date, nothing is known about the implications of such an extreme social and breeding system on the immune function of its members.

Using a wild population of meerkats, our first objective was to test the hypothesis that the energetic demands of maintaining social status constrain investment in innate immune function. If so, our main prediction is that dominant animals of both sexes should be more immunosuppressed than subordinates. Thereafter, we sought to determine if variation in immune function related to other correlates of status, including body condition, age and sex hormone concentrations. Lastly, using a subset of animals, we also looked for changes in immune function associated with reproductive variables. For males, we compared the immune function of rovers (i.e. subordinate males that temporarily leave the clan to pursue extra-clan mating opportunities [45]) to that of non-rovers; for females, we compared the immune function of pregnant females to that of non-pregnant females.

Our additional non-mutually exclusive predictions were as follows: If parasitism serves as a proxy for innate immune function in meerkats, as has been demonstrated in some (e.g. African buffalo, *Syncerus caffer* [46]), but not in other (e.g. whitefooted mice, *Peromyscus leucopus*, and prairie voles, *Microtus ochrogaster* [47]) species, we would expect the immunosuppression of dominant individuals to be consistent with their increased parasitism [23]. Owing to the covariance between dominance and age, a similar status-related pattern could reflect an age-related decline in immune function or ‘immunosenescence’ [48]. In this scenario, age might be more reliable than status as a predictor of immune function. Moreover, if androgens were broadly immunosuppressive, we would predict immunological differences between social classes in females, but not in males. The cumulative effects of energetic constraints, immunosenescence and androgens could contribute to immunosuppression in dominant animals and produce status-related differences in innate immunity that would be especially pronounced in females. Nevertheless, we might expect the opposite status-related pattern if any immunosuppressive correlates of dominance were overshadowed by the immunosuppressive effects of poor body condition in subordinates. Lastly, if reproduction were traded off against immune function [49], we would expect immunosuppression in dominant individuals and also in reproductively active subordinates (i.e. roving males and pregnant females). Given that T concentrations are raised in males during extra-territorial roving [22,50] and that A_4_ and T concentrations increase in females over gestation [22], such trade-offs might be mediated by androgens.

## 2. Methods

### 2.1. Study site and subjects

We conducted this study, from September 2014 to March 2015 (i.e. from spring through summer), on a wild population of meerkats in the Kuruman River Reserve (KRR) (26°58′ S, 21°49′ E), an area comprising sparsely vegetated sand dunes and herbaceous flats in South Africa's Kalahari Desert. The study site, habitat and climate have been described elsewhere [51]. Individual meerkats were identifiable by unique dye marks and were habituated to the presence of humans, which allowed for routine weighing and close observation (less than 1 m). Thus, we knew all relevant life-history variables at the time of sampling.

Our focal subjects derived from 19 groups and included 75 sexually mature meerkats (which are presented by sex and dominance status in table 1). We classified males as roving if they had been absent from the group in the 2 days before or after sample collection [22]. We classified females as pregnant at the time of sampling based on weight gain and abdominal swelling [33].

**Table 1.** Mean weight, age, BKA, HCA and steroid hormone concentrations for dominant and subordinate meerkats of both sexes. Numbers under each subject category represent individuals first (in italics), followed by sample numbers.

	mean (s.e.) value by subject category			
	dominant male	subordinate male	dominant female	subordinate female
variable	*9*/11	*28*/30	*11*/15	*27*/36
weight (g)	733.500 (19.746)	643.767 (15.698)	689.500 (19.666)	628.097 (13.642)
age (month)	51.843 (3.995)	27.955 (3.574)	36.685 (4.957)	20.336 (1.906)
BKA (% killed)	41.105 (5.135)	44.450 (3.390)	34.955 (2.011)	56.921 (4.044)
HCA (CH50)	221.219 (18.441)	209.154 (8.706)	178.033 (17.320)	232.102 (5.996)
A_4_ (ng ml^−1^)	1.533 (0.294)	2.764 (0.651)	27.718 (4.775)	3.148 (0.563)
T (ng ml^−1^)	7.872 (2.528)	11.629 (2.355)	14.488 (5.076)	5.667 (0.930)
E_2_ (pg ml^−1^)	402.496 (57.279)	477.964 (74.248)	1502.474 (378.855)	606.160 (66.682)

Both tuberculosis (TB [52]) and gastrointestinal parasites [23,53] are prevalent within this meerkat population, with an estimated 6% of meerkats dying from TB [54]. Because the immune response can be altered during infections, we excluded from our study all meerkats with signs of TB; however, because over 95% of meerkats harbour gastrointestinal parasites, we assumed that all of our subjects were concurrently parasitized.

### 2.2. Sampling procedures

Animal capture, blood collection and serum processing procedures have been provided previously [22]. Briefly, we captured individual meerkats upon emergence from the sleeping burrow in the morning, anaesthetized them with isoflurane (Isofor; Safe Line Pharmaceuticals, Johannesburg, South Africa) and collected 0.2–2 ml of blood from the jugular vein, which we immediately transferred to serum separator tubes (Vacutainer^®^, Becton Dickinson, Franklin Lakes, NJ, USA). We processed the samples and stored the serum, in 0.075–0.200 ml aliquots, on site at −80°C until transport, on dry ice, to Duke University in Durham, North Carolina, where we kept them at −80°C until analysis. The time from sample collection to analysis varied from 3 to 11 months, but this time difference had no effect on the integrity of our immune measures (see below).

### 2.3. Innate immune measures

To quantify innate immunity, we used a sterile environment to perform two related, but distinct, assays of serum, including a bacteria-killing assay (BKA) and a haemolytic complement assay (HCA). For each assay, we used one aliquot per sample and ensured that no aliquots had been previously thawed. We ran all samples in triplicate.

We measured the ability of complement proteins and natural antibodies to lyse a known quantity of *Escherichia coli* (ATTC #8739) using the microplate method of the BKA developed by French & Neuman-Lee [28]. We optimized the assay by diluting serum 1 : 30, as this was the sample dilution that yielded approximately 50% killing. We calculated the bacterial killing ability of serum as the percentage of bacteria killed relative to positive controls.

Using the HCA methods outlined by Demas *et al.* [27] and Sinclair & Lochmiller [55], we determined the ability of complement to induce haemolysis of a foreign antigen (i.e. sheep red blood cells). Prior to performing the assay, we optimized dilutions of serum and used the dilutions, 1 : 150 and 1 : 300, that resulted in 30–70% haemolysis. We express haemolytic-complement activity in CH_50_ units or the reciprocal of the dilution that causes 50% haemolysis [56].

We confirmed that the integrity of our immune measures was not compromised following long-term storage of serum: We found no difference in same-sample BKA scores (*t*_9_ = 0.292; *p* = 0.777) or HCA scores (*t*_9_ = −0.570; *p* = 0.583) following an additional year of storage at −80°C, and both immune measures were highly correlated between years (BKA: 0.787; *t*_8_ = 3.612; *p* = 0.007; HCA: 0.890; *t*_8_ = 5.534; *p* < 0.001).

### 2.4. Hormone assays

We used a subset of serum samples that we had previously analysed for A_4_, T and E_2_ [22]. Briefly, we ran samples in duplicate using commercial, competitive enzyme immunoassay kits (ALPCO diagnostics, Salem, NH, USA). We re-analysed samples for which coefficients of variation (CV) exceeded 10%. Details about assay validation are provided elsewhere [22]: The A_4_ assay has a sensitivity of 0.04 ng ml^−1^ using a 25 µl dose, with an intra- and inter-assay CV of 5.23% and 8.7%, respectively. Cross reactivity of the A_4_ assay was 1.8% with dehydroepiandrosterone (DHEA), 0.2% with T, less than 0.1% with oestrone, E_2_, progesterone, 17-OH progesterone and 5α-dihydrotestosterone (DHT), less than 0.01% with cortisol and DHEA sulphate (DHEA-S). The T assay has a sensitivity of 0.02 ng ml^−1^ using a 50 µl dose, with an intra- and inter-assay CV of 7.9% and 7.3%, respectively. Cross reactivity of the T assay was 5.2% with DHT, 1.4% with A_4_, 0.8% with androstanediol, 0.5% with progesterone, 0.1% with androsterone and less than 0.1% with aldosterone, andrenosterone, cholesterol, corticosterone, DHEA, DHEA-S, epiandrosterone, E_2_, oestriol and pregnenolone. The E_2_ assay has a sensitivity of 10 pg ml^−1^ using a 50 µl dose, with an intra- and inter-assay CV of 7.7% and 8.7%, respectively. Cross reactivity of the E_2_ assay was 1.6% with oestriol, 1.3% with oestrone and 0.1% with progesterone and cortisol.

### 2.5. Statistical analyses

To test for sex and status differences in immune measures, we ran a series of linear mixed models (LMMs) with the following explanatory variables: sex (two categories: male and female), social status (two categories: dominant and subordinate), body condition (initially as a continuous variable of body condition index and, subsequently, as a continuous variable of body weight in grams; see below), age (continuous variable in months) and the interaction between sex and social status. We examined the relationships between sex steroids (A_4_, T and E_2_) and immune responses by re-running the models with each hormone, plus all possible two-way and three-way interactions involving sex, social status and the hormone of interest.

To test if reproductive variables were related to immune function in males, we compared BKA and HCA scores of 11 subordinate rovers to those of their non-roving counterparts. To test if reproductive variables were related to immune function in females, we compared BKA and HCA scores of 11 pregnant subordinates to their non-pregnant counterparts. We focused on subordinate females because, with the exception of two individuals, dominant females were frequently pregnant. We included a reproductive variable (two categories: roving or non-roving; pregnant or non-pregnant), subject weight and age as fixed effects.

For our measure of body condition, we initially estimated a body condition index or ‘BCI’ for each individual and included BCI, instead of weight, as an explanatory variable. We calculated BCI as the residuals of the log-transformed weight regressed against the hindfoot length, a proxy for total body length. We estimated BCI separately for pregnant and non-pregnant individuals because the relationship between weight and hindfoot length varied depending on whether or not an animal was pregnant. Although we initially included BCI in our models, BCI was never a significant predictor of immune function and so we opted to substitute weight, a proxy of body condition, for BCI. This substitution enabled us to maximize our sample size because, unlike hindfoot measures, which were acquired last during procedures and could not always be obtained, we determined weights for all individuals.

For all models, we included the individual nested within its clan as a random effect, used log-transformed BKA scores and fit the models using maximum likelihood. We examined all possible combinations of our fixed effects, scaled all continuous variables (i.e. for each continuous variable, we subtracted the mean and divided by the standard deviation) and compared models using the corrected Akaike information criterion (AICc [57]). Because analyses based on AIC can yield several acceptable models, we used a model-averaging approach when one or more models showed substantial support (i.e. ΔAICc ≤ 2) [57]. All models within 2 AICc (hereafter ‘top models’) were considered equally acceptable [57]. We evaluated the relative importance of fixed effects by averaging parameter estimates across the top models [57] and determined significance by examining 95% confidence intervals (CIs) and *z*-score statistics. In instances with only one top model, we determined significance using a likelihood ratio test.

We assessed the assumption of normally distributed residuals by visually inspecting quantile–quantile plots for all models. We verified the assumption of homogeneity of variances by examining plots of standardized residuals versus fitted values. We assessed colinearity between all main effects by calculating variance inflation factors (VIFs). Because all VIFs were under 2, we retained all variables [58,59]. When final models yielded significant interactions, we made *post hoc* comparisons using Tukey's test; however, *post hoc* tests were not possible for averaged models. Because small serum volumes occasionally limited our ability to measure all immune and endocrine parameters in all individuals, we present sample sizes with model results.

We used program R version 3.3.1 [60] for all statistical analyses. We used the *nlme* package for mixed effects models [61] and the *MuMIn* package for AICc model selection [62].

## 3. Results

### 3.1. Social correlates of immune function

**Figure 1.**
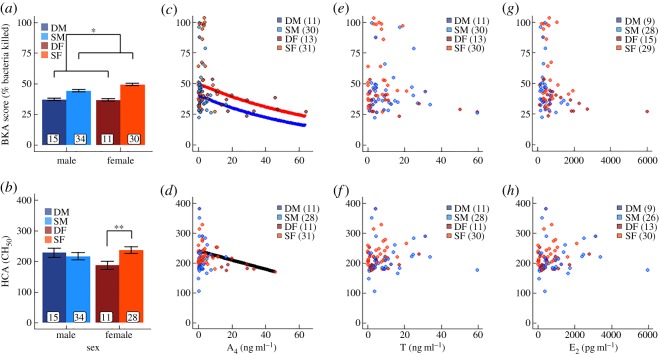
Dominant meerkats (males: dark blue; females: dark red) generally have weaker immune responses than do subordinates (males: light blue; females: light red). Shown by sex and social status in the first column are the predicted means ± s.e.m. for bacteria-killing assay (BKA) scores (*a*,*c*,*e*,*g*) and haemolytic complement assay (HCA) scores (*b*,*d*,*f*,*h*) derived from the top model(s) (see electronic supplementary material, tables S2–S9). Sample sizes are included at the bottom of the bar graphs or in the legends. Shown in columns 2–4 are relationships between immune measures and androstenedione (A_4_; column 2), testosterone (T; column 3), and oestradiol (E_2_; column 4). Lines (males: blue; females: red; both sexes: black) show significant relationships predicted by the top models (table 2). **p < 0.01, *p < 0.05.

Social status was an important predictor of BKA scores (in both sexes) and HCA scores (in females only), and dominant females had the lowest immune responses (table 1). Dominant animals, regardless of sex, had lower BKA scores than did subordinates (figure 1; tables 1 and 2; for all candidate models see electronic supplementary material, tables S2–S9). Consistent with status effects for BKA scores, dominant females had reduced HCA scores compared to subordinate females (*p* = 0.003; figure 1; tables 1 and 2; for all candidate models see electronic supplementary material, tables S2–S9); however, social status was not predictive of HCA scores in males (figure 1; tables 1 and 2; for all candidate models see electronic supplementary material, tables S2–S9). There was also a significant interaction between sex and social status for HCA scores (table 2), but it was largely driven by a single, dominant female that had the lowest complement activity. Upon removing this individual from our analyses, the interaction between sex and status was suggestive, but no longer statistically significant (*p* = 0.075, *n.s.*; electronic supplementary material, table S1). Nevertheless, dominant females still had significantly lower HCA scores than did subordinate females (*p* = 0.010; *t*_54_ = −3.166). Although dominant individuals were heavier and older than were subordinates (table 1), we could detect no relationship between either weight or age and immunocompetence (table 2).

**Table 2.** Factors associated with innate immune function in wild meerkats, as determined by multimodel weighted average analysis. All predictor variables initially included in the full models are listed as ‘starting predictors’, whereas those that were retained in the top model(s) are listed as ‘predictors’. For each predictor, importance was calculated as the sum of AICc weights over all models in which the predictor was present. All comparisons were made against the indicated levels of each factor (status = dominant, sex = female). Total sample sizes are indicated for each model (see figure 1 for sample size breakdowns by sex and status).

response	starting predictors (*n*)	predictor	estimate	lower 95% CI	upper 95% CI	statistic^a^	*p*	importance
BKA	sex × status + weight + age (90)	*intercept*	*3*.*602*	*3*.*415*	*3*.*789*	*37*.*705*	<*0*.*001*	—
		sex	0.019	−0.295	0.333	0.118	0.906	0.68
		*status*	*0*.*296*	*0*.*057*	*0*.*535*	*2*.*430*	*0*.*015*	*1*.*00*
		sex : status	−0.302	−0.634	0.030	1.780	0.075	0.41
	A_4_ × sex × status + weight + age (85)	*intercept*	*3*.*830*	*3*.*641*	*4*.*019*	*39*.*757*	<*0*.*001*	—
		*A_4_*	*−0*.*138*	*−0*.*257*	*−0*.*020*	*2*.*288*	*0*.*022*	*1*.*00*
		*sex*	*−0*.*220*	*−0*.*406*	*−0*.*035*	*2*.*328*	*0*.*020*	*1*.*00*
		status	0.145	−0.062	0.352	1.373	0.170	0.42
		weight	−0.050	−0.156	0.056	0.931	0.352	0.21
		A_4_ : sex	−0.252	−0.811	0.308	0.883	0.378	0.14
	T × sex × status + weight + age (84)	*intercept*	*3*.*603*	*3*.*401*	*3*.*805*	*34*.*929*	<*0*.*001*	—
		sex	−0.009	−0.327	0.345	0.052	0.958	0.59
		*status*	*0*.*292*	*0*.*030*	*0*.*555*	*2*.*183*	*0*.*029*	*1*.*00*
		sex : status	−0.318	−0.676	0.039	1.746	0.081	0.35
		T	−0.418	−0.142	0.058	0.821	0.412	0.13
	E_2_ × sex × status + weight + age (81)	*intercept*	*3*.*630*	*3*.*423*	*3*.*836*	*34*.*445*	<*0*.*001*	—
		sex	0.016	−0.350	0.383	0.087	0.931	0.70
		*status*	*0*.*269*	*0*.*005*	*0*.*533*	*2*.*000*	*0*.*046*	*1*.*00*
		sex : status	−0.322	−0.704	0.060	1.652	0.099	0.43
		E_2_	−0.043	−0.135	0.049	0.912	0.362	0.49
		E_2_ : sex	−0.170	−0.455	0.114	1.173	0.241	0.14
		weight	−0.039	−0.161	0.084	0.618	0.537	0.12
		E_2_ : status	−0.119	−0.338	0.100	1.062	0.288	0.06
HCA	sex × status + weight + age (88)	*intercept*	*187*.*545*	*160*.*486*	*214*.*605*	—	—	—
		sex	40.630	3.325	77.935	0.012^b^	0.912	*1*.*00*
		status	49.193	18.756	79.630	2.921^b^	0.087	*1*.*00*
		*sex : status*	*−60*.*418*	*−60*.*418*	*−15*.*957*	*6*.*938*^b^	*0*.*008*	*1*.*00*
	A_4_ × sex × status + weight + age (81)	*intercept*	*230*.*781*	*209*.*561*	*252*.*00*	*21*.*316*	<*0*.*001*	—
		*A_4_*	*−17*.*979*	*−33*.*687*	*−2*.*272*	*2*.*243*	*0*.*025*	*1*.*00*
		sex	−13.720	−35.587	8.146	1.230	0.219	0.76
		A_4_ : sex	30.157	−27.627	87.940	1.023	0.306	0.29
	T × sex × status + weight + age (80)	*intercept*	*227*.*530*	*202*.*632*	*252*.*427*	*17*.*912*	<*0*.*001*	—
		sex	−11.242	−36.758	14.275	0.864	0.388	0.58
		age	−4.792	−14.065	4.480	1.013	0.311	0.19
		status	15.718	−9.895	41.331	1.203	0.229	0.28
		T	6.468	−6.733	19.688	0.960	0.337	0.09
		sex : status	−27.200	−65.173	10.772	1.404	0.160	0.08
		weight	−4.642	−16.867	7.583	0.744	0.457	0.14
	E_2_ × sex × status + weight + age (78)	*intercept*	*224*.*691*	*202*.*857*	*246*.*524*	*20*.*170*	<*0*.*001*	—
		age	−4.983	−14.236	4.271	1.055	0.291	0.24
		status	10.562	−9.679	30.804	1.023	0.306	0.21
		sex	−7.729	−25.163	9.704	0.869	0.385	0.18

^a^*Z* scores from averaged models.

^b^Likelihood ratio test statistic.

### 3.2. Endocrine correlates of immune function

Certain hormone concentrations, however, were significantly linked to immune function. Notably, A_4_, but not T or E_2_, proved to be the best predictor of innate immunity (figure 1 and table 2; for all candidate models see electronic supplementary material, tables S2–S9): BKA and HCA scores decreased with increasing A_4_ concentrations. Moreover, the relationship between A_4_ and immune function transcended an individual's social status, as social status was no longer significant after including A_4_ in our models. Yet, for the HCA model, the inclusion of A_4_ revealed a female bias in HCA scores (figure 1 and table 2). For the HCA models involving hormones, we did not include the two samples from the aforementioned dominant female because she was an outlier. Inclusion of those two samples produced a significant negative relationship between HCA scores and T in dominant females (*χ*^2^ = 9.587; *p* = 0.002)—a relationship that was otherwise non-significant (table 2). This outlier dominant female acquired dominance in late 2013 and, since that time, consistently has had low immune responses, as well as raised T concentrations (second only to a roving male in our study).

### 3.3. Immune function during reproductive events

Although reproductive activity could shift energy away from immune defence, we did not detect differences in immunocompetence between roving and non-roving subordinate males (BKA: *F*_1, 26_ = 0.981; *p* = 0.331; HCA: *F*_1, 24_ = 1.895; *p* = 0.181) or between pregnant and non-pregnant subordinate females (BKA: *χ*^2^ = 0.409; *p* = 0.523; HCA: *χ*^2^ = 0.559; *p* = 0.455).

## 4. Discussion

Understanding the causes of individual variation in immune function is a central goal of ecological immunologists, and investigators are just beginning to appreciate how social organization and individual social status mediate immunity in group-living animals. Here, in the female-dominant, cooperatively breeding meerkat, we report that dominant animals experience an immunocompromise, which is more pronounced within females than within males. Although dominant meerkats were in better condition and older than subordinates, we could detect no relation between body condition or age and immunocompetence. We also found no evidence that reproductive activity was related to immune function. Instead, A_4_, but not T or E_2_, emerged as the best predictor of innate immunity: For all animals, regardless of social status, innate immune function decreased with increasing A_4_ concentrations. Because A_4_ is also a correlate of female dominance in meerkats [22], an endocrine mechanism may underlie trade-offs between social status and immune function in this species.

Although status-related differences in susceptibility to parasites and recovery from injuries have been documented in a variety of species (greenfinch, *Carduelis chloris* [63]; house finch, *Carpodacus mexicanus* [64]; chimpanzee, *Pan troglodytes schweinfurthii* [16]; yellow baboon, *Papio cynocephalus* [65,66]), including meerkats [23,24], it is less clear if immunological mechanisms mediate these vulnerabilities. Because, in this study, dominant meerkats had a lower innate immune response than did subordinates, their greater gastrointestinal parasite burdens probably derive from enhanced susceptibility to infection. Thus, parasite burdens can be an appropriate proxy for immune function. That said, the strength of the immune response may not always correlate positively to or be indicative of individual health. For instance, a negative correlation may exist between resistance (i.e. reducing parasite or pathogen burden) and tolerance (i.e. limiting the damage caused) [67,68]. A robust immune response may increase resistance to pathogens, but may concurrently increase the risk of immunopathology. Conversely, although immunosuppression could come at the expense of resistance, it may be adaptive if it limits immunopathological damage [69]. Maintaining a low level of innate immune function also could be beneficial if it allows for investment in the components of immunity most relevant to survival. For instance, meerkats might benefit from investing in adaptive immunity, at the expense of innate immunity, to protect against *Mycobacterium suricattae*. This bacterium is the causative pathogen of meerkat TB, which is prevalent within the population and highly pathogenic [52]. Such trade-offs between innate and adaptive immunity are widespread, and the optimal balance of investment in different components of immunity probably depends on a variety of factors, including local ecological conditions, life stage and social status [70,71].

The effects of social status on immunity are unlikely to function in isolation, but instead may act synergistically with other factors, such as reproduction. Upon acquiring dominance, male and female meerkats experience energetic costs that could further limit their immune function: notably, males engage in energetically expensive bouts of scent marking and mate guarding [39], and females, beyond engaging in dominance assertions and aggressively targeting subordinate dams [72], are frequently pregnant [32,33,41,73]. Yet, consistent with immunocompetence in other species [74,75] and with parasitism in meerkats [23], we found no evidence that pregnancy, *per se*, was linked to immunosuppression. Our analysis, however, was limited to subordinate females, so we were unable to determine if relationships between immune function and pregnancy differed by social status. For dominant females, which reproduce at greater rates than do subordinates and often reconceive within mere days of parturition [73], it is possible that the cumulative effects of frequent reproduction (e.g. elevated GC concentrations ([76] but see [33]); the energetic demands of lactation [77]) limit their immune responsiveness.

Meerkats are characterized by a female bias in A_4_ and by the absence of a sex difference in T [22], both of which are extremely unusual patterns among mammals [78–81]. To the best of our knowledge, the only other species exhibiting a sex reversal in A_4_ is the spotted hyaena [81,82]. In addition to their hormonal masculinization, female spotted hyaenas are dominant over males [83] and thus make an interesting comparison to meerkats. As in meerkats, T does not appear to be linked to immune function in hyaenas [9]; however, in contrast to patterns in the meerkat, immune function in spotted hyaenas increases with social rank and is greatest in high-ranking females. In a species characterized by such extreme food competition [83], perhaps priority of access to large, non-monopolizable resources buffers dominant female hyaenas from immunological trade-offs [9]. By contrast, the small, monopolizable food resources of meerkats may minimize the impact of food competition [33] on their immune function. Moreover, in spotted hyaenas, rank in a linear dominance hierarchy is maternally acquired [83,84], whereas in meerkats, individuals compete for breeding status in a despotic society [32]. The opposite status-related, immunity patterns observed in the females of these species may relate to differences in their manner of rank acquisition and in the costs and benefits of dominance. Across species, female dominance alone cannot explain class differences in immune function; other factors, such as feeding ecology and social organization, may also be relevant.

Sex could be an additional factor affecting immunocompetence: Immune function is generally greater in females than in males, a pattern that has been attributed, in part, to sex differences in hormone concentrations [85]. In meerkats, however, sex was not a consistent predictor of immune function, perhaps because this species shows minimal sex differences in either androgens or oestrogens. In contrast to intersexual comparisons, within female meerkats hormone concentrations vary by social status and may be involved in mediating their intrasexual differences in immune function.

In the immunocompetence handicap hypothesis (ICHH), Folstad & Karter [13] proposed a proximate endocrine mechanism to explain how variation in secondary sexual characteristics could honestly signal male quality. A key aspect of this hypothesis relates variation in T concentrations (such as might be associated with differences in social status) to male parasitism and immune function. Notably, the authors suggested that T, for example, could be a double-edged sword promoting reproductive success (via enhanced expression of ornaments) at the cost of reduced immunocompetence. Since then, based on either directly measured or inferred T concentrations, the ICHH has been invoked to explain selection for costly secondary sex characteristics in males [15,16]; however, support for the hypothesis has been equivocal [86]. Inconsistencies across studies may owe to the fact that, over time, the ICHH has been inexorably (and perhaps sometimes incorrectly) linked to T-mediated immunosuppression, specifically. Nevertheless, an important and often overlooked part of the ICHH is the proposal, by Folstad & Karter [13], that the trade-off could be mediated by any self-regulated biochemical substance that has the dualistic effect of stimulating trait expression and compromising immune function (i.e. not necessarily always by T). Similarly overlooked is the potential utility of the ICHH to explain trade-offs, not only in males, but also in females [24].

For most vertebrates, T is an appealing hormone to consider because masculinity is generally associated with androgens; however, T probably interacts with other hormones to influence immune function, and, thus, endocrine-immune relationships may not be evident from measuring T alone. A_4_ is a putatively weak androgen (or prohormone) and a precursor to both T and E_2_, but, in meerkats, it was the most reliable predictor of immune function, accounting for immunological differences between social classes. Although little is known about A_4_'s immunomodulatory effects, it may influence the immune system via local, enzymatic conversion to either T or E_2_. If so, one might have expected a correlation between T or E_2_ and immunosuppression, which we failed to detect. Although these null findings do not preclude the involvement of these hormones, they may implicate androgenic precursors more directly. Therefore, a second, non-mutually exclusive possibility is that A_4_ is immunosuppressive in its own right, exerting its effects via binding to androgen receptors [87,88] or via non-genomic pathways (i.e. independent of androgen receptors) [89]. As in other species, the meerkat androgen receptor could be sensitive to A_4_, a proposal that remains to be tested.

Similar to our findings on A_4_ in meerkats, other androgens besides T have been shown to be relevant in other species: For instance, the androgen precursor dehydroepiandrosterone (DHEA) has immunomodulating effects in orangutans (*Pongo pygmaeus morio* [90]) and in humans (*Homo sapiens* [91]). In some teleost species, such as the three-spined stickleback (*Gasterosteus aculeatus*), the dominant circulating androgen is not T, but 11-ketotestosterone (11-kT) and, in males, concentrations of 11-kT (but not T) correlate positively with ornamentation and negatively with immunocompetence [92]. For invertebrates that lack androgens or androgen-signalling pathways, yet other hormones may be involved in the trade-off between sexual traits and immunocompetence. For instance, juvenile hormone, which influences aspects of insect behaviour and physiology, mediates the ICHH in damselflies (*Hetaerina americana* [93]). Therefore, looking beyond the actions of T in males to explore other biochemical mediators of immunosuppression, specifically, or the ICHH more broadly, in both sexes, may reveal complex endocrine-immune dynamics that could lead to new insights regarding the drivers of health disparities and the evolution of sexually selected traits.

## Supplementary Material

Model Selection and Averaging Tables

## Data accessibility

The datasets supporting this manuscript have been uploaded as part of the electronic supplementary material.
