# Supplementary material for: Social and endocrine correlates of immune function in meerkats: implications for the immunocompetence handicap hypothesis
Source: R Soc Open Sci. 2018 Aug 1;5(8):180435. doi: 10.1098/rsos.180435 (PMC6124081; doi:10.1098/rsos.180435)
Supplement: Model Selection and Averaging Tables [file rsos180435supp1.docx]

**Supplementary Material**

**Social and endocrine correlates of immune function in meerkats: implications for the immunocompetence handicap hypothesis**

Kendra N. Smyth, Nicholas M. Caruso, Charli S. Davies, Tim H. Clutton-Brock and Christine M. Drea

Supplemental Table 1: Factors associated with complement activity in meerkats

Supplemental Tables 2-9: Model selection tables for immune measures with and without hormones

**Supplemental Table 1.** Factors associated with hemolytic complement activity in wild meerkats after removing an outlying dominant female from the analysis. All predictor variables initially included in the full model are listed as ‘starting predictors’, whereas those that were retained in the top model(s) are listed as ‘predictors.’ For each predictor, importance was calculated as the sum of AICc weights over all models in which the predictor was present. All comparisons were made against the indicated levels of each factor (status = dominant, sex = female).

^a^ Z scores from averaged models.

| **response** | **starting**  **predictors (*n*)** | **predictor** | **estimate** | **lower 95% CI** | **upper 95% CI** | **statistic^a^** | ***p*** | **importance** |
| --- | --- | --- | --- | --- | --- | --- | --- | --- |
| HCA | sex x status +  weight + age (86) | *intercept* | *221.868* | *196.589* | *247.148* | *17.202* | *< 0.001* | — |
|  |  | sex | -1.494 | -34.872 | 31.883 | 0.088 | 0.930 | 0.48 |
|  |  | status | 20.479 | -5.990 | 46.948 | 1.516 | 0.129 | 0.45 |
|  |  | sex:status | -33.880 | -71.120 | 3.359 | 1.783 | 0.075 | 0.18 |
|  |  | age | -5.303 | -14.504 | 3.898 | 1.130 | 0.259 | 0.20 |
|  |  | weight | -4.131 | -16.436 | 8.175 | 0.658 | 0.511 | 0.07 |

**Supplemental Table 2.** Model selection (AICc) table for BKA (*n* = 90 observations). Italicized terms indicate the top models (ΔAICc < 2). Initial parameters include sex x status, age and weight.

| **fixed effects** | ***df*** | **log likelihood** | **AICc** | **ΔAICc** | **weight** |
| --- | --- | --- | --- | --- | --- |
| *BKA ~ intercept + sex x* *status* | *7* | *-30.027* | *75.420* | *0.000* | *0.214* |
| *BKA ~ intercept + status* | *5* | *-32.588* | *75.891* | *0.471* | *0.169* |
| *BKA ~ intercept + sex + status* | *6* | *-31.643* | *76.298* | *0.878* | *0.138* |
| BKA ~ intercept + age + sex x status | 8 | -29.912 | 77.602 | 2.182 | 0.072 |
| BKA ~ intercept + weight + sex x status | 8 | -29.922 | 77.623 | 2.202 | 0.071 |
| BKA ~ intercept + status + weight | 6 | -32.528 | 78.068 | 2.648 | 0.057 |
| BKA ~ intercept + status + age | 6 | -32.579 | 78.170 | 2.750 | 0.054 |
| BKA ~ intercept + sex + status + age | 7 | -31.462 | 78.290 | 2.870 | 0.051 |
| BKA ~ intercept + sex + status + weight | 7 | -31.613 | 78.593 | 3.172 | 0.044 |
| BKA ~ intercept + age + weight + sex x status | 9 | -29.613 | 79.475 | 4.055 | 0.028 |
| BKA ~ intercept + status + age + weight | 7 | -32.464 | 80.294 | 4.874 | 0.019 |
| BKA ~ intercept + sex + status + age + weight | 8 | -31.280 | 80.337 | 4.917 | 0.018 |
| BKA ~ intercept | 4 | -36.118 | 80.707 | 5.287 | 0.015 |
| BKA ~ intercept + weight | 5 | -35.197 | 81.108 | 5.687 | 0.012 |
| BKA ~ intercept + sex | 5 | -35.355 | 81.425 | 6.004 | 0.011 |
| BKA ~ intercept + age | 5 | -35.572 | 81.858 | 6.438 | 0.009 |
| BKA ~ intercept + sex + weight | 6 | -34.541 | 82.093 | 6.673 | 0.008 |
| BKA ~ intercept + sex + age | 6 | -35.071 | 83.154 | 7.734 | 0.004 |
| BKA ~ intercept + age + weight | 6 | -35.145 | 83.302 | 7.882 | 0.004 |
| BKA ~ intercept + sex + age + weight | 7 | -34.540 | 84.445 | 9.025 | 0.002 |

**Supplemental Table 3.** Model selection (AICc) table for HCA (*n* = 88 observations). Italicized terms indicate the top models (ΔAICc < 2). Initial parameters include sex x status, age and weight.

| **fixed effects** | **df** | **log likelihood** | **AICc** | **ΔAICc** | **weight** |
| --- | --- | --- | --- | --- | --- |
| *HCA ~ intercept + sex x status* | *7* | *-461.971* | *939.342* | *0.000* | *0.329* |
| HCA ~ intercept + status | 5 | -465.446 | 941.624 | 2.282 | 0.105 |
| HCA ~ intercept + weight + sex x status | 8 | -461.904 | 941.632 | 2.290 | 0.105 |
| HCA ~ intercept + age + sex x status | 8 | -461.967 | 941.758 | 2.415 | 0.098 |
| HCA ~ intercept | 4 | -466.907 | 942.296 | 2.953 | 0.075 |
| HCA ~ intercept + status + weight | 6 | -465.185 | 943.408 | 4.065 | 0.043 |
| HCA ~ intercept + status + age | 6 | -465.423 | 943.884 | 4.541 | 0.034 |
| HCA ~ intercept + sex + status | 6 | -465.440 | 943.917 | 4.575 | 0.033 |
| HCA ~ intercept + weight + age + sex x status | 9 | -461.898 | 944.103 | 4.761 | 0.030 |
| HCA ~ intercept + age | 5 | -466.748 | 944.228 | 4.886 | 0.029 |
| HCA ~ intercept + sex | 5 | -466.901 | 944.533 | 5.191 | 0.025 |
| HCA ~ intercept + weight | 5 | -466.905 | 944.541 | 5.199 | 0.024 |
| HCA ~ intercept + status + weight + age | 7 | -465.171 | 945.741 | 6.399 | 0.013 |
| HCA ~ intercept + sex + status + weight | 7 | -465.176 | 945.751 | 6.409 | 0.013 |
| HCA ~ intercept + sex + status + age | 7 | -465.406 | 946.212 | 6.869 | 0.011 |
| HCA ~ intercept + weight + age | 6 | -466.696 | 946.430 | 7.088 | 0.009 |
| HCA ~ intercept + sex + age | 6 | -466.747 | 946.531 | 7.189 | 0.009 |
| HCA ~ intercept + sex + weight | 6 | -466.899 | 946.834 | 7.492 | 0.008 |
| HCA ~ intercept + sex + status + weight + age | 8 | -465.167 | 948.157 | 8.815 | 0.004 |
| HCA ~ intercept + sex + weight + age | 7 | -466.691 | 948.782 | 9.440 | 0.003 |

**Supplemental Table 4.** Model selection (AICc) table for BKA with A_4_ (*n* = 85 observations). Italicized terms indicate the top models (ΔAICc < 2). Initial parameters include A_4_ x sex x status, age and weight.

| **fixed effects** | ***df*** | **log likelihood** | **AICc** | **ΔAICc** | **weight** |
| --- | --- | --- | --- | --- | --- |
| *BKA ~ intercept + A_4_ + sex* | *6* | *-30.227* | *73.531* | *0.000* | *0.115* |
| *BKA ~ intercept + A_4_ + sex + status* | *7* | *-29.318* | *74.090* | *0.559* | *0.087* |
| *BKA ~ intercept + A_4_ + sex + weight* | *7* | *-29.612* | *74.679* | *1.147* | *0.065* |
| *BKA ~ intercept + status + A_4_ x sex* | *8* | *-28.805* | *75.505* | *1.974* | *0.043* |
| BKA ~ intercept + A_4_ + sex + age | 7 | -30.069 | 75.593 | 2.061 | 0.041 |
| BKA ~ intercept + A_4_ x sex | 7 | -30.096 | 75.646 | 2.114 | 0.040 |
| BKA ~ intercept + sex + A_4_ x status | 8 | -28.997 | 75.888 | 2.357 | 0.035 |
| BKA ~ intercept + A_4_ + sex + status + weight | 8 | -29.109 | 76.113 | 2.582 | 0.032 |
| BKA ~ intercept + sex x status | 7 | -30.378 | 76.210 | 2.678 | 0.030 |
| BKA ~ intercept + A_4_ + sex x status | 8 | -29.160 | 76.214 | 2.683 | 0.030 |
| BKA ~ intercept + weight + A_4_ x sex | 8 | -29.271 | 76.437 | 2.906 | 0.027 |
| BKA ~ intercept + A_4_ + sex + status + age | 8 | -29.312 | 76.518 | 2.987 | 0.026 |
| BKA ~ intercept + A_4_ + status | 6 | -31.917 | 76.911 | 3.380 | 0.021 |
| BKA ~ intercept + status | 5 | -33.114 | 76.988 | 3.456 | 0.020 |
| BKA ~ intercept + A_4_ + sex + weight + age | 8 | -29.611 | 77.117 | 3.586 | 0.019 |
| BKA ~ intercept + sex + status | 6 | -32.031 | 77.138 | 3.607 | 0.019 |
| BKA ~ intercept + A_4_ | 5 | -33.297 | 77.353 | 3.821 | 0.017 |
| BKA ~ intercept + status + weight + A_4_ x sex | 9 | -28.492 | 77.384 | 3.853 | 0.017 |
| BKA ~ intercept + age + A_4_ x sex | 8 | -29.817 | 77.530 | 3.998 | 0.016 |
| BKA ~ intercept + A_4_ x sex + sex x status | 9 | -28.600 | 77.599 | 4.068 | 0.015 |
| BKA ~ intercept + A_4_ x status + sex x status | 9 | -28.646 | 77.692 | 4.161 | 0.014 |
| BKA ~ intercept + sex + weight + A_4_ x status | 9 | -28.731 | 77.862 | 4.331 | 0.013 |
| BKA ~ intercept + A_4_ x sex + A_4_ x status | 9 | -28.776 | 77.953 | 4.422 | 0.013 |
| BKA ~ intercept + A_4_ x status | 7 | -31.267 | 77.989 | 4.457 | 0.012 |
| BKA ~ intercept + status + age + A_4_ x sex | 9 | -28.803 | 78.007 | 4.475 | 0.012 |
| BKA ~ intercept + A_4_ + weight | 6 | -32.507 | 78.092 | 4.560 | 0.012 |
| BKA ~ intercept + A_4_ + weight + sex x status | 9 | -28.924 | 78.248 | 4.717 | 0.011 |
| BKA ~ intercept + weight + sex x status | 8 | -30.210 | 78.314 | 4.783 | 0.011 |
| BKA ~ intercept + A_4_ + age | 6 | -32.634 | 78.346 | 4.814 | 0.010 |
| BKA ~ intercept + sex + age + A_4_ x status | 9 | -28.996 | 78.391 | 4.860 | 0.010 |
| BKA ~ intercept + A_4_ + sex + status + weight + age | 9 | -29.011 | 78.422 | 4.890 | 0.010 |
| BKA ~ intercept + age + sex x status | 8 | -30.321 | 78.536 | 5.005 | 0.009 |
| BKA ~ intercept + A_4_ + age + sex x status | 9 | -29.152 | 78.703 | 5.172 | 0.009 |
| BKA ~ intercept + A_4_ + status + weight | 7 | -31.707 | 78.869 | 5.338 | 0.008 |
| BKA ~ intercept + weight + age + A_4_ x sex | 9 | -29.266 | 78.932 | 5.401 | 0.008 |
| BKA ~ intercept + status + weight | 6 | -33.027 | 79.130 | 5.599 | 0.007 |
| BKA ~ intercept + A_4_ + status + age | 7 | -31.849 | 79.153 | 5.622 | 0.007 |
| BKA ~ intercept + status + age | 6 | -33.114 | 79.305 | 5.773 | 0.006 |
| BKA ~ intercept + sex + status + age | 7 | -31.933 | 79.322 | 5.790 | 0.006 |
| BKA ~ intercept + sex + status + weight | 7 | -31.972 | 79.399 | 5.868 | 0.006 |
| BKA ~ intercept + weight + A_4_ x sex + sex x status | 10 | -28.241 | 79.455 | 5.923 | 0.006 |
| BKA ~ intercept + weight + A_4_ x status + sex x status | 10 | -28.302 | 79.577 | 6.046 | 0.006 |
| BKA ~ intercept + status + weight + age + A_4_ x sex | 10 | -28.439 | 79.850 | 6.319 | 0.005 |
| BKA ~ intercept + weight + A_4_ x sex + A_4_ x status | 10 | -28.458 | 79.890 | 6.358 | 0.005 |
| BKA ~ intercept + weight + A_4_ x status | 8 | -31.010 | 79.916 | 6.384 | 0.005 |
| BKA ~ intercept + A_4_ x sex + A_4_ x status + sex x status | 10 | -28.492 | 79.956 | 6.425 | 0.005 |
| BKA ~ intercept + age + A_4_ x sex + sex x status | 10 | -28.599 | 80.171 | 6.639 | 0.004 |
| BKA ~ intercept + A_4_ + weight + age | 7 | -32.366 | 80.187 | 6.655 | 0.004 |
| BKA ~ intercept + age + A_4_ x status | 8 | -31.173 | 80.241 | 6.709 | 0.004 |
| BKA ~ intercept + sex + weight + age + A_4_ x status | 10 | -28.643 | 80.260 | 6.728 | 0.004 |
| BKA ~ intercept + age + A_4_ x status + sex x status | 10 | -28.644 | 80.261 | 6.730 | 0.004 |
| BKA ~ intercept + weight + age + sex x status | 9 | -29.978 | 80.355 | 6.824 | 0.004 |
| BKA ~ intercept + age + A_4_ x sex + A_4_ x status | 10 | -28.775 | 80.523 | 6.991 | 0.003 |
| BKA ~ intercept + A_4_ + weight + age + sex x status | 10 | -28.806 | 80.584 | 7.053 | 0.003 |
| BKA ~ intercept | 4 | -36.343 | 81.185 | 7.654 | 0.002 |
| BKA ~ intercept + A_4_ + status + weight + age | 8 | -31.705 | 81.304 | 7.773 | 0.002 |
| BKA ~ intercept + sex + status + weight + age | 8 | -31.741 | 81.378 | 7.846 | 0.002 |
| BKA ~ intercept + status + weight + age | 7 | -32.995 | 81.444 | 7.912 | 0.002 |
| BKA ~ intercept + weight | 5 | -35.430 | 81.620 | 8.088 | 0.002 |
| BKA ~ intercept + sex | 5 | -35.466 | 81.691 | 8.160 | 0.002 |
| BKA ~ intercept + weight + A_4_ x sex + A_4_ x status + sex x status | 11 | -28.107 | 81.831 | 8.300 | 0.002 |
| BKA ~ intercept + weight + age + A_4_ x sex + sex x status | 11 | -28.167 | 81.951 | 8.420 | 0.002 |
| BKA ~ intercept + weight + age + A_4_ x status + sex x status | 11 | -28.182 | 81.980 | 8.449 | 0.002 |
| BKA ~ intercept + age | 5 | -35.699 | 82.157 | 8.626 | 0.002 |
| BKA ~ intercept + A_4_ x sex x status | 11 | -28.295 | 82.207 | 8.675 | 0.002 |
| BKA ~ intercept + sex + weight | 6 | -34.628 | 82.333 | 8.801 | 0.001 |
| BKA ~ intercept + weight + age + A_4_ x status | 9 | -31.004 | 82.408 | 8.876 | 0.001 |
| BKA ~ intercept + weight + age + A_4_ x sex + A_4_ x status | 11 | -28.403 | 82.422 | 8.890 | 0.001 |
| BKA ~ intercept + age + A_4_ x sex + A_4_ x status + sex x status | 11 | -28.491 | 82.599 | 9.068 | 0.001 |
| BKA ~ intercept + sex + age | 6 | -35.085 | 83.247 | 9.715 | 0.001 |
| BKA ~ intercept + weight + age | 6 | -35.329 | 83.735 | 10.204 | 0.001 |
| BKA ~ intercept + weight + A_4_ x sex x status | 12 | -27.944 | 84.222 | 10.690 | 0.001 |
| BKA ~ intercept + weight + age + A_4_ x sex + A_4_ x status + sex x status | 12 | -28.022 | 84.377 | 10.846 | 0.001 |
| BKA ~ intercept + sex + weight + age | 7 | -34.611 | 84.676 | 11.144 | 0.000 |
| BKA ~ intercept + age + A_4_ x sex x status | 12 | -28.290 | 84.913 | 11.382 | 0.000 |
| BKA ~ intercept + weight + age + A_4_ x sex x status | 13 | -27.891 | 86.909 | 13.378 | 0.000 |

**Supplemental Table 5.** Model selection (AICc) table for HCA with A_4_ (*n* =81 observations). Italicized terms indicate the top models (ΔAICc < 2). Initial parameters include A_4_ x sex x status, age and weight.

| **fixed effects** | ***df*** | **log likelihood** | **AICc** | **ΔAICc** | **weight** |
| --- | --- | --- | --- | --- | --- |
| *HCA ~ intercept + A_4_ + sex* | *6* | *-412.620* | *838.375* | *0.000* | *0.152* |
| *HCA ~ intercept + A_4_ x sex* | *7* | *-411.900* | *839.335* | *0.961* | *0.094* |
| *HCA ~ intercept + A_4_* | *5* | *-414.445* | *839.689* | *1.315* | *0.079* |
| HCA ~ intercept + A_4_ + sex + age | 7 | -412.492 | 840.517 | 2.143 | 0.052 |
| HCA ~ intercept + A_4_ + sex + weight | 7 | -412.604 | 840.743 | 2.368 | 0.047 |
| HCA ~ intercept + A_4_ + sex + status | 7 | -412.619 | 840.772 | 2.397 | 0.046 |
| HCA ~ intercept + A_4_ + age | 6 | -414.011 | 841.157 | 2.782 | 0.038 |
| HCA ~ intercept + status + A_4_ x sex | 8 | -411.777 | 841.554 | 3.180 | 0.031 |
| HCA ~ intercept + age + A_4_ x sex | 8 | -411.882 | 841.764 | 3.389 | 0.028 |
| HCA ~ intercept + weight + A_4_ x sex | 8 | -411.892 | 841.785 | 3.410 | 0.028 |
| HCA ~ intercept + A_4_ + status | 6 | -414.382 | 841.898 | 3.524 | 0.026 |
| HCA ~ intercept + A_4_ + weight | 6 | -414.440 | 842.015 | 3.641 | 0.025 |
| HCA ~ intercept | 4 | -416.952 | 842.430 | 4.056 | 0.020 |
| HCA ~ intercept + sex + A_4_ x status | 8 | -412.303 | 842.606 | 4.231 | 0.018 |
| HCA ~ intercept + A_4_ + sex + age + status | 8 | -412.429 | 842.858 | 4.484 | 0.016 |
| HCA ~ intercept + A_4_ + sex + age + weight | 8 | -412.475 | 842.950 | 4.576 | 0.015 |
| HCA ~ intercept + status | 5 | -416.097 | 842.993 | 4.618 | 0.015 |
| HCA ~ intercept + A_4_ + sex x status | 8 | -412.562 | 843.125 | 4.750 | 0.014 |
| HCA ~ intercept + A_4_ + sex + weight + status | 8 | -412.594 | 843.188 | 4.814 | 0.014 |
| HCA ~ intercept + A_4_ + age + weight | 7 | -413.845 | 843.224 | 4.849 | 0.013 |
| HCA ~ intercept + sex x status | 7 | -413.927 | 843.389 | 5.014 | 0.012 |
| HCA ~ intercept + sex | 5 | -416.343 | 843.487 | 5.112 | 0.012 |
| HCA ~ intercept + A_4_ + age + status | 7 | -413.996 | 843.526 | 5.152 | 0.012 |
| HCA ~ intercept + age | 5 | -416.436 | 843.672 | 5.297 | 0.011 |
| HCA ~ intercept + age + status + A_4_ x sex | 9 | -411.660 | 843.856 | 5.482 | 0.010 |
| HCA ~ intercept + A_4_ x sex + sex x status | 9 | -411.763 | 844.062 | 5.687 | 0.009 |
| HCA ~ intercept + A_4_ x sex + A_4_ x status | 9 | -411.771 | 844.077 | 5.702 | 0.009 |
| HCA ~ intercept + weight + status + A_4_ x sex | 9 | -411.772 | 844.080 | 5.705 | 0.009 |
| HCA ~ intercept + A_4_ x status | 7 | -414.295 | 844.123 | 5.749 | 0.009 |
| HCA ~ intercept + age + weight + A_4_ x sex | 9 | -411.836 | 844.206 | 5.832 | 0.008 |
| HCA ~ intercept + sex + status | 6 | -415.536 | 844.207 | 5.832 | 0.008 |
| HCA ~ intercept + A_4_ + weight + status | 7 | -414.379 | 844.292 | 5.918 | 0.008 |
| HCA ~ intercept + weight | 5 | -416.880 | 844.559 | 6.185 | 0.007 |
| HCA ~ intercept + sex + age + A_4_ x status | 9 | -412.147 | 844.829 | 6.455 | 0.006 |
| HCA ~ intercept + sex + weight + A_4_ x status | 9 | -412.291 | 845.118 | 6.743 | 0.005 |
| HCA ~ intercept + A_4_ x status + sex x status | 9 | -412.302 | 845.139 | 6.764 | 0.005 |
| HCA ~ intercept + age + status | 6 | -416.008 | 845.152 | 6.778 | 0.005 |
| HCA ~ intercept + sex + age | 6 | -416.016 | 845.166 | 6.792 | 0.005 |
| HCA ~ intercept + weight + status | 6 | -416.065 | 845.266 | 6.891 | 0.005 |
| HCA ~ intercept + A_4_ + age + sex x status | 9 | -412.391 | 845.318 | 6.944 | 0.005 |
| HCA ~ intercept + A_4_ + sex + age + weight + status | 9 | -412.424 | 845.383 | 7.008 | 0.005 |
| HCA ~ intercept + A_4_ + weight + sex x status | 9 | -412.530 | 845.595 | 7.220 | 0.004 |
| HCA ~ intercept + sex + weight | 6 | -416.234 | 845.602 | 7.228 | 0.004 |
| HCA ~ intercept + A_4_ + age + weight + status | 8 | -413.843 | 845.686 | 7.312 | 0.004 |
| HCA ~ intercept + age + sex x status | 8 | -413.896 | 845.792 | 7.418 | 0.004 |
| HCA ~ intercept + weight + sex x status | 8 | -413.915 | 845.829 | 7.455 | 0.004 |
| HCA ~ intercept + age + A_4_ x status | 8 | -413.925 | 845.850 | 7.475 | 0.004 |
| HCA ~ intercept + age + weight | 6 | -416.395 | 845.925 | 7.550 | 0.003 |
| HCA ~ intercept + age + weight + status + A_4_ x sex | 10 | -411.647 | 846.436 | 8.062 | 0.003 |
| HCA ~ intercept + age + A_4_ x sex + sex x status | 10 | -411.653 | 846.449 | 8.074 | 0.003 |
| HCA ~ intercept + age + A_4_ x sex + A_4_ x status | 10 | -411.655 | 846.453 | 8.079 | 0.003 |
| HCA ~ intercept + sex + age + status | 7 | -415.517 | 846.568 | 8.193 | 0.003 |
| HCA ~ intercept + weight + A_4_ x status | 8 | -414.287 | 846.574 | 8.200 | 0.003 |
| HCA ~ intercept + sex + weight + status | 7 | -415.525 | 846.585 | 8.211 | 0.003 |
| HCA ~ intercept + weight + A_4_ x sex + sex x status | 10 | -411.756 | 846.655 | 8.281 | 0.002 |
| HCA ~ intercept + A_4_ x sex + A_4_ x status + sex x status | 10 | -411.762 | 846.666 | 8.291 | 0.002 |
| HCA ~ intercept + weight + A_4_ x sex + A_4_ x status | 10 | -411.766 | 846.675 | 8.301 | 0.002 |
| HCA ~ intercept + age + weight + status | 7 | -415.874 | 847.282 | 8.908 | 0.002 |
| HCA ~ intercept + sex + age + weight + A_4_ x status | 10 | -412.136 | 847.415 | 9.041 | 0.002 |
| HCA ~ intercept + age + A_4_ x status + sex x status | 10 | -412.147 | 847.437 | 9.062 | 0.002 |
| HCA ~ intercept + sex + age + weight | 7 | -416.014 | 847.562 | 9.188 | 0.002 |
| HCA ~ intercept + weight + A_4_ x status + sex x status | 10 | -412.289 | 847.721 | 9.347 | 0.001 |
| HCA ~ intercept + A_4_ + age + weight + sex x status | 10 | -412.390 | 847.923 | 9.548 | 0.001 |
| HCA ~ intercept + age + weight + A_4_ x status | 9 | -413.746 | 848.027 | 9.653 | 0.001 |
| HCA ~ intercept + age + weight + sex x status | 9 | -413.896 | 848.326 | 9.952 | 0.001 |
| HCA ~ intercept + sex + age + weight + status | 8 | -415.476 | 848.951 | 10.576 | 0.001 |
| HCA ~ intercept + age + weight + A_4_ x sex + A_4_ x status | 11 | -411.641 | 849.108 | 10.733 | 0.001 |
| HCA ~ intercept + age + weight + A_4_ x sex + sex x status | 11 | -411.642 | 849.111 | 10.736 | 0.001 |
| HCA ~ intercept + age + A_4_ x sex + A_4_ x status + sex x status | 11 | -411.651 | 849.128 | 10.753 | 0.001 |
| HCA ~ intercept + A_4_ x sex x status | 11 | -411.735 | 849.296 | 10.922 | 0.001 |
| HCA ~ intercept + weight + A_4_ x sex + A_4_ x status + sex x status | 11 | -411.755 | 849.337 | 10.962 | 0.001 |
| HCA ~ intercept + age + weight + A_4_ x status + sex x status | 11 | -412.136 | 850.097 | 11.723 | 0.000 |
| HCA ~ intercept + age + A_4_ x sex x status | 12 | -411.622 | 851.833 | 13.458 | 0.000 |
| HCA ~ intercept + age + weight + A_4_ x sex + A_4_ x status + sex x status | 12 | -411.639 | 851.866 | 13.492 | 0.000 |
| HCA ~ intercept + weight + A_4_ x sex x status | 12 | -411.730 | 852.048 | 13.674 | 0.000 |
| HCA ~ intercept + age + weight + A_4_ x sex x status | 13 | -411.607 | 854.648 | 16.273 | 0.000 |

**Supplemental Table 6.** Model selection (AICc) table for BKA with T (*n* = 84 observations). Italicized terms indicate the top models (ΔAICc < 2). Initial parameters include T x sex x status, age and weight.

| **fixed effects** | ***df*** | **log likelihood** | **AICc** | **ΔAICc** | **weight** |
| --- | --- | --- | --- | --- | --- |
| *BKA ~ intercept + sex x status* | *7* | *-30.080* | *75.634* | *0.000* | *0.123* |
| *BKA ~ intercept + status* | *5* | *-32.669* | *76.108* | *0.473* | *0.097* |
| *BKA ~ intercept + sex + status* | *6* | *-31.631* | *76.353* | *0.719* | *0.086* |
| *BKA ~ intercept + status + T* | *6* | *-32.245* | *77.582* | *1.947* | *0.047* |
| BKA ~ intercept + age + sex x status | 8 | -29.919 | 77.759 | 2.124 | 0.043 |
| BKA ~ intercept + T + sex x status | 8 | -29.962 | 77.843 | 2.209 | 0.041 |
| BKA ~ intercept + weight + sex x status | 8 | -29.999 | 77.919 | 2.285 | 0.039 |
| BKA ~ intercept + sex + status + T | 7 | -31.289 | 78.051 | 2.416 | 0.037 |
| BKA ~ intercept + status + weight | 6 | -32.601 | 78.294 | 2.659 | 0.033 |
| BKA ~ intercept + sex + status + age | 7 | -31.448 | 78.370 | 2.735 | 0.031 |
| BKA ~ intercept + status + age | 6 | -32.656 | 78.404 | 2.769 | 0.031 |
| BKA ~ intercept + sex + status + weight | 7 | -31.589 | 78.652 | 3.018 | 0.027 |
| BKA ~ intercept + status + T + weight | 7 | -32.138 | 79.750 | 4.116 | 0.016 |
| BKA ~ intercept | 4 | -35.624 | 79.754 | 4.120 | 0.016 |
| BKA ~ intercept + age + weight + sex x status | 9 | -29.661 | 79.755 | 4.121 | 0.016 |
| BKA ~ intercept + status x T | 7 | -32.241 | 79.955 | 4.320 | 0.014 |
| BKA ~ intercept + status + T + age | 7 | -32.244 | 79.962 | 4.328 | 0.014 |
| BKA ~ intercept + sex x status + status x T | 9 | -29.810 | 80.053 | 4.419 | 0.014 |
| BKA ~ intercept + T + age + sex x status | 9 | -29.836 | 80.105 | 4.470 | 0.013 |
| BKA ~ intercept + weight | 5 | -34.679 | 80.128 | 4.493 | 0.013 |
| BKA ~ intercept + T + weight + sex x status | 9 | -29.848 | 80.128 | 4.494 | 0.013 |
| BKA ~ intercept + sex + status x T | 8 | -31.150 | 80.219 | 4.585 | 0.012 |
| BKA ~ intercept + sex + status + T + age | 8 | -31.160 | 80.240 | 4.606 | 0.012 |
| BKA ~ intercept + sex | 5 | -34.781 | 80.331 | 4.697 | 0.012 |
| BKA ~ intercept + sex + status + T + weight | 8 | -31.207 | 80.335 | 4.700 | 0.012 |
| BKA ~ intercept + sex x status + sex x T | 9 | -29.961 | 80.354 | 4.720 | 0.012 |
| BKA ~ intercept + sex + status + age + weight | 8 | -31.255 | 80.430 | 4.796 | 0.011 |
| BKA ~ intercept + status + sex x T | 8 | -31.277 | 80.475 | 4.840 | 0.011 |
| BKA ~ intercept + status + age + weight | 7 | -32.527 | 80.527 | 4.893 | 0.011 |
| BKA ~ intercept + sex + weight | 6 | -33.944 | 80.980 | 5.345 | 0.009 |
| BKA ~ intercept + age | 5 | -35.114 | 80.997 | 5.363 | 0.008 |
| BKA ~ intercept + T | 5 | -35.154 | 81.077 | 5.443 | 0.008 |
| BKA ~ intercept + T + weight | 6 | -34.088 | 81.266 | 5.632 | 0.007 |
| BKA ~ intercept + sex + T | 6 | -34.391 | 81.874 | 6.240 | 0.005 |
| BKA ~ intercept + status + T + age + weight | 8 | -32.090 | 82.100 | 6.466 | 0.005 |
| BKA ~ intercept + sex + age | 6 | -34.508 | 82.106 | 6.472 | 0.005 |
| BKA ~ intercept + T + age + weight + sex x status | 10 | -29.563 | 82.139 | 6.505 | 0.005 |
| BKA ~ intercept + weight + status x T | 8 | -32.129 | 82.178 | 6.544 | 0.005 |
| BKA ~ intercept + T + age | 6 | -34.545 | 82.182 | 6.547 | 0.005 |
| BKA ~ intercept + sex + status + T + age + weight | 9 | -30.938 | 82.309 | 6.674 | 0.004 |
| BKA ~ intercept + age + weight | 6 | -34.643 | 82.377 | 6.743 | 0.004 |
| BKA ~ intercept + sex x status + status x T + sex x T | 10 | -29.689 | 82.392 | 6.757 | 0.004 |
| BKA ~ intercept + weight + sex x status + status x T | 10 | -29.693 | 82.400 | 6.765 | 0.004 |
| BKA ~ intercept + age + status x T | 8 | -32.240 | 82.400 | 6.766 | 0.004 |
| BKA ~ intercept + sex + T + weight | 7 | -33.476 | 82.425 | 6.791 | 0.004 |
| BKA ~ intercept + age + sex x status + status x T | 10 | -29.725 | 82.464 | 6.830 | 0.004 |
| BKA ~ intercept + sex + age + status x T | 9 | -31.058 | 82.548 | 6.914 | 0.004 |
| BKA ~ intercept + sex + weight + status x T | 9 | -31.068 | 82.569 | 6.935 | 0.004 |
| BKA ~ intercept + status x T + sex x T | 9 | -31.108 | 82.648 | 7.014 | 0.004 |
| BKA ~ intercept + age + sex x status + sex x T | 10 | -29.836 | 82.686 | 7.052 | 0.004 |
| BKA ~ intercept + weight + sex x status + sex x T | 10 | -29.847 | 82.707 | 7.073 | 0.004 |
| BKA ~ intercept + status + age + sex x T | 9 | -31.155 | 82.741 | 7.107 | 0.004 |
| BKA ~ intercept + status + weight + sex x T | 9 | -31.194 | 82.820 | 7.186 | 0.003 |
| BKA ~ intercept + sex + age + weight | 7 | -33.943 | 83.359 | 7.725 | 0.003 |
| BKA ~ intercept + T + age + weight | 7 | -34.036 | 83.546 | 7.912 | 0.002 |
| BKA ~ intercept + sex + T + age | 7 | -34.048 | 83.569 | 7.935 | 0.002 |
| BKA ~ intercept + sex x status x T | 11 | -29.085 | 83.838 | 8.203 | 0.002 |
| BKA ~ intercept + sex x T | 7 | -34.212 | 83.897 | 8.263 | 0.002 |
| BKA ~ intercept + weight + sex x T | 8 | -33.317 | 84.555 | 8.920 | 0.001 |
| BKA ~ intercept + age + weight + status x T | 9 | -32.087 | 84.606 | 8.972 | 0.001 |
| BKA ~ intercept + age + weight + sex x status + status x T | 11 | -29.473 | 84.613 | 8.979 | 0.001 |
| BKA ~ intercept + sex + age + weight + status x T | 10 | -30.859 | 84.732 | 9.098 | 0.001 |
| BKA ~ intercept + age + weight + sex x status + sex x T | 11 | -29.563 | 84.792 | 9.158 | 0.001 |
| BKA ~ intercept + weight + sex x status + status x T + sex x T | 11 | -29.589 | 84.846 | 9.211 | 0.001 |
| BKA ~ intercept + sex + T + age + weight | 8 | -33.464 | 84.847 | 9.213 | 0.001 |
| BKA ~ intercept + status + age + weight + sex x T | 10 | -30.932 | 84.877 | 9.243 | 0.001 |
| BKA ~ intercept + age + sex x status + status x T + sex x T | 11 | -29.613 | 84.893 | 9.258 | 0.001 |
| BKA ~ intercept + age + status x T + sex x T | 10 | -31.021 | 85.056 | 9.421 | 0.001 |
| BKA ~ intercept + weight + status x T + sex x T | 10 | -31.035 | 85.083 | 9.449 | 0.001 |
| BKA ~ intercept + age + sex x T | 8 | -33.877 | 85.674 | 10.040 | 0.001 |
| BKA ~ intercept + weight + sex x status x T | 12 | -28.980 | 86.355 | 10.720 | 0.001 |
| BKA ~ intercept + age + sex x status x T | 12 | -28.989 | 86.373 | 10.738 | 0.001 |
| BKA ~ intercept + age + weight + sex x T | 9 | -33.306 | 87.044 | 11.410 | 0.000 |
| BKA ~ intercept + age + weight + sex x status + status x T + sex x T | 12 | -29.393 | 87.180 | 11.545 | 0.000 |
| BKA ~ intercept + age + weight + status x T + sex x T | 11 | -30.837 | 87.342 | 11.707 | 0.000 |
| BKA ~ intercept + age + weight + sex x status x T | 13 | -28.743 | 88.687 | 13.052 | 0.000 |

**Supplemental Table 7.** Model selection (AICc) table for HCA with T (*n* = 80 observations). Italicized terms indicate the top models (ΔAICc < 2). Initial parameters include T x sex x status, age and weight.

| **fixed effects** | ***df*** | **log likelihood** | **AICc** | **ΔAICc** | **weight** |
| --- | --- | --- | --- | --- | --- |
| *HCA ~ intercept + sex* | *5* | *-410.326* | *831.463* | *0.000* | *0.081* |
| *HCA ~ intercept* | *4* | *-411.508* | *831.549* | *0.086* | *0.078* |
| *HCA ~ intercept + age* | *5* | *-410.705* | *832.222* | *0.759* | *0.056* |
| *HCA ~ intercept + status* | *5* | *-410.787* | *832.385* | *0.922* | *0.051* |
| *HCA ~ intercept + sex + status* | *6* | *-409.667* | *832.485* | *1.022* | *0.049* |
| *HCA ~ intercept + sex + T* | *6* | *-409.735* | *832.621* | *1.158* | *0.046* |
| *HCA ~ intercept + sex x status* | *7* | *-408.606* | *832.768* | *1.306* | *0.042* |
| *HCA ~ intercept + sex + age* | *6* | *-409.840* | *832.831* | *1.368* | *0.041* |
| *HCA ~ intercept + sex + weight* | *6* | *-409.901* | *832.952* | *1.489* | *0.039* |
| *HCA ~ intercept + weight* | *5* | *-411.209* | *833.229* | *1.767* | *0.034* |
| HCA ~ intercept + T + sex x status | 8 | -407.753 | 833.533 | 2.070 | 0.029 |
| HCA ~ intercept + T | 5 | -411.401 | 833.613 | 2.150 | 0.028 |
| HCA ~ intercept + sex + status + T | 7 | -409.121 | 833.798 | 2.335 | 0.025 |
| HCA ~ intercept + age + status | 6 | -410.505 | 834.160 | 2.697 | 0.021 |
| HCA ~ intercept + sex + T + weight | 7 | -409.362 | 834.280 | 2.817 | 0.020 |
| HCA ~ intercept + sex + age + T | 7 | -409.447 | 834.450 | 2.987 | 0.018 |
| HCA ~ intercept + age + T | 6 | -410.663 | 834.478 | 3.015 | 0.018 |
| HCA ~ intercept + status + T | 6 | -410.693 | 834.537 | 3.074 | 0.017 |
| HCA ~ intercept + age + weight | 6 | -410.705 | 834.561 | 3.099 | 0.017 |
| HCA ~ intercept + sex x T | 7 | -409.559 | 834.673 | 3.210 | 0.016 |
| HCA ~ intercept + sex + age + status | 7 | -409.567 | 834.690 | 3.227 | 0.016 |
| HCA ~ intercept + status + weight | 6 | -410.770 | 834.692 | 3.229 | 0.016 |
| HCA ~ intercept + sex + status + weight | 7 | -409.597 | 834.749 | 3.286 | 0.016 |
| HCA ~ intercept + weight + sex x status | 8 | -408.463 | 834.953 | 3.490 | 0.014 |
| HCA ~ intercept + age + sex x status | 8 | -408.515 | 835.059 | 3.596 | 0.013 |
| HCA ~ intercept + sex + age + weight | 7 | -409.772 | 835.100 | 3.637 | 0.013 |
| HCA ~ intercept + T + weight | 6 | -411.129 | 835.408 | 3.945 | 0.011 |
| HCA ~ intercept + T + weight + sex x status | 9 | -407.622 | 835.816 | 4.353 | 0.009 |
| HCA ~ intercept + sex x status + sex x T | 9 | -407.650 | 835.872 | 4.409 | 0.009 |
| HCA ~ intercept + status + sex x T | 8 | -408.986 | 836.001 | 4.538 | 0.008 |
| HCA ~ intercept + sex x status + status x T | 9 | -407.720 | 836.011 | 4.549 | 0.008 |
| HCA ~ intercept + sex + status x T | 8 | -408.996 | 836.020 | 4.557 | 0.008 |
| HCA ~ intercept + age + T + sex x status | 9 | -407.747 | 836.065 | 4.602 | 0.008 |
| HCA ~ intercept + sex + status + T + weight | 8 | -409.068 | 836.163 | 4.701 | 0.008 |
| HCA ~ intercept + sex + age + status + T | 8 | -409.102 | 836.232 | 4.769 | 0.007 |
| HCA ~ intercept + weight + sex x T | 8 | -409.185 | 836.398 | 4.935 | 0.007 |
| HCA ~ intercept + age + status + T | 7 | -410.451 | 836.458 | 4.995 | 0.007 |
| HCA ~ intercept + age + sex x T | 8 | -409.231 | 836.490 | 5.027 | 0.007 |
| HCA ~ intercept + age + status + weight | 7 | -410.485 | 836.525 | 5.062 | 0.006 |
| HCA ~ intercept + status x T | 7 | -410.519 | 836.593 | 5.130 | 0.006 |
| HCA ~ intercept + sex + age + T + weight | 8 | -409.331 | 836.690 | 5.227 | 0.006 |
| HCA ~ intercept + age + T + weight | 7 | -410.663 | 836.882 | 5.419 | 0.005 |
| HCA ~ intercept + status + T + weight | 7 | -410.682 | 836.920 | 5.457 | 0.005 |
| HCA ~ intercept + sex + age + status + weight | 8 | -409.553 | 837.134 | 5.671 | 0.005 |
| HCA ~ intercept + age + weight + sex x status | 9 | -408.448 | 837.466 | 6.004 | 0.004 |
| HCA ~ intercept + sex x T + status x T | 9 | -408.751 | 838.073 | 6.610 | 0.003 |
| HCA ~ intercept + weight + sex x status + sex x T | 10 | -407.510 | 838.208 | 6.745 | 0.003 |
| HCA ~ intercept + sex x status + sex x T + status x T | 10 | -407.565 | 838.318 | 6.855 | 0.003 |
| HCA ~ intercept + weight + sex x status + status x T | 10 | -407.579 | 838.347 | 6.884 | 0.003 |
| HCA ~ intercept + sex x status x T | 11 | -406.252 | 838.387 | 6.924 | 0.003 |
| HCA ~ intercept + age + T + weight + sex x status | 10 | -407.603 | 838.394 | 6.931 | 0.003 |
| HCA ~ intercept + status + weight + sex x T | 9 | -408.925 | 838.422 | 6.959 | 0.003 |
| HCA ~ intercept + sex + weight + status x T | 9 | -408.927 | 838.425 | 6.962 | 0.002 |
| HCA ~ intercept + age + sex x status + sex x T | 10 | -407.635 | 838.459 | 6.996 | 0.002 |
| HCA ~ intercept + age + status + sex x T | 9 | -408.949 | 838.470 | 7.007 | 0.002 |
| HCA ~ intercept + sex + age + status x T | 9 | -408.984 | 838.540 | 7.077 | 0.002 |
| HCA ~ intercept + age + sex x status + status x T | 10 | -407.716 | 838.621 | 7.158 | 0.002 |
| HCA ~ intercept + age + status x T | 8 | -410.318 | 838.664 | 7.202 | 0.002 |
| HCA ~ intercept + sex + age + status + T + weight | 9 | -409.067 | 838.706 | 7.243 | 0.002 |
| HCA ~ intercept + age + weight + sex x T | 9 | -409.135 | 838.842 | 7.379 | 0.002 |
| HCA ~ intercept + age + status + T + weight | 8 | -410.430 | 838.889 | 7.426 | 0.002 |
| HCA ~ intercept + weight + status x T | 8 | -410.497 | 839.022 | 7.559 | 0.002 |
| HCA ~ intercept + weight + sex x T + status x T | 10 | -408.661 | 840.511 | 9.048 | 0.001 |
| HCA ~ intercept + age + sex x T + status x T | 10 | -408.723 | 840.634 | 9.171 | 0.001 |
| HCA ~ intercept + weight + sex x status + sex x T + status x T | 11 | -407.403 | 840.688 | 9.225 | 0.001 |
| HCA ~ intercept + age + weight + sex x status + sex x T | 11 | -407.501 | 840.885 | 9.422 | 0.001 |
| HCA ~ intercept + weight + sex x status x T | 12 | -406.129 | 840.914 | 9.451 | 0.001 |
| HCA ~ intercept + age + weight + sex x status + status x T | 11 | -407.549 | 840.981 | 9.518 | 0.001 |
| HCA ~ intercept + age + sex x status + sex x T + status x T | 11 | -407.553 | 840.988 | 9.525 | 0.001 |
| HCA ~ intercept + age + status + weight + sex x T | 10 | -408.920 | 841.028 | 9.565 | 0.001 |
| HCA ~ intercept + sex + age + weight + status x T | 10 | -408.925 | 841.038 | 9.575 | 0.001 |
| HCA ~ intercept + age + sex x status x T | 12 | -406.210 | 841.077 | 9.614 | 0.001 |
| HCA ~ intercept + age + weight + status x T | 9 | -410.311 | 841.193 | 9.731 | 0.001 |
| HCA ~ intercept + age + weight + sex x T + status x T | 11 | -408.661 | 843.205 | 11.742 | 0.000 |
| HCA ~ intercept + age + weight + sex x status + sex x T + status x T | 12 | -407.386 | 843.428 | 11.966 | 0.000 |
| HCA ~ intercept + age + weight + sex x status x T | 13 | -406.129 | 843.772 | 12.310 | 0.000 |

**Supplemental Table 8.** Model selection (AICc) table for BKA with E_2_ (*n* = 81 observations). Italicized terms indicate the top models (ΔAICc < 2). Initial parameters include E_2_ x sex x status, age and weight.

| **fixed effects** | ***df*** | **log likelihood** | **AICc** | **ΔAICc** | **weight** |
| --- | --- | --- | --- | --- | --- |
| *BKA ~ intercept + sex x status* | *7* | *-28.323* | *72.180* | *0.000* | *0.068* |
| *BKA ~ intercept + status* | *5* | *-30.695* | *72.189* | *0.009* | *0.067* |
| *BKA ~ intercept + status + E_2_* | *6* | *-29.947* | *73.028* | *0.848* | *0.044* |
| *BKA ~ intercept + sex + status + E_2_* | *7* | *-28.747* | *73.029* | *0.849* | *0.044* |
| *BKA ~ intercept + E_2_ + sex x status* | *8* | *-27.523* | *73.045* | *0.865* | *0.044* |
| *BKA ~ intercept + sex + status* | *6* | *-30.016* | *73.167* | *0.987* | *0.041* |
| *BKA ~ intercept + status + sex x E_2_* | *8* | *-27.797* | *73.593* | *1.413* | *0.033* |
| *BKA ~ intercept + sex x status + sex x E_2_* | *9* | *-26.606* | *73.747* | *1.568* | *0.031* |
| *BKA ~ intercept + weight + sex x status* | *8* | *-27.996* | *73.993* | *1.813* | *0.027* |
| *BKA ~ intercept + status + weight* | *6* | *-30.492* | *74.118* | *1.939* | *0.026* |
| *BKA ~ intercept + sex x status + status x E_2_* | *9* | *-26.809* | *74.154* | *1.974* | *0.025* |
| BKA ~ intercept + status + age | 6 | -30.694 | 74.522 | 2.342 | 0.021 |
| BKA ~ intercept + E_2_ | 5 | -31.864 | 74.527 | 2.347 | 0.021 |
| BKA ~ intercept + age + sex x status | 8 | -28.280 | 74.560 | 2.381 | 0.021 |
| BKA ~ intercept + status x E_2_ | 7 | -29.558 | 74.650 | 2.470 | 0.020 |
| BKA ~ intercept + sex + status x E_2_ | 8 | -28.373 | 74.745 | 2.566 | 0.019 |
| BKA ~ intercept | 4 | -33.113 | 74.753 | 2.573 | 0.019 |
| BKA ~ intercept + E_2_ + weight + sex x status | 9 | -27.205 | 74.946 | 2.766 | 0.017 |
| BKA ~ intercept + sex + E_2_ | 6 | -30.906 | 74.947 | 2.767 | 0.017 |
| BKA ~ intercept + status + E_2_ + weight | 7 | -29.728 | 74.991 | 2.811 | 0.017 |
| BKA ~ intercept + sex + status + E_2_ + weight | 8 | -28.558 | 75.115 | 2.935 | 0.016 |
| BKA ~ intercept + sex + status + weight | 7 | -29.836 | 75.205 | 3.026 | 0.015 |
| BKA ~ intercept + weight | 5 | -32.203 | 75.206 | 3.027 | 0.015 |
| BKA ~ intercept + E_2_ + weight | 6 | -31.047 | 75.229 | 3.050 | 0.015 |
| BKA ~ intercept + status + E_2_ + age | 7 | -29.927 | 75.387 | 3.207 | 0.014 |
| BKA ~ intercept + sex + status + age | 7 | -29.938 | 75.411 | 3.231 | 0.013 |
| BKA ~ intercept + sex + status + E_2_ + age | 8 | -28.731 | 75.462 | 3.282 | 0.013 |
| BKA ~ intercept + E_2_ + age + sex x status | 9 | -27.515 | 75.565 | 3.385 | 0.012 |
| BKA ~ intercept + weight + sex x status + sex x E_2_ | 10 | -26.276 | 75.695 | 3.515 | 0.012 |
| BKA ~ intercept + status + weight + sex x E_2_ | 9 | -27.603 | 75.741 | 3.561 | 0.011 |
| BKA ~ intercept + sex + E_2_ + weight | 7 | -30.105 | 75.745 | 3.565 | 0.011 |
| BKA ~ intercept + E_2_ + age | 6 | -31.354 | 75.844 | 3.664 | 0.011 |
| BKA ~ intercept + weight + sex x status + status x E_2_ | 10 | -26.360 | 75.863 | 3.683 | 0.011 |
| BKA ~ intercept + weight + age + sex x status | 9 | -27.684 | 75.904 | 3.724 | 0.010 |
| BKA ~ intercept + sex x status + sex x E_2_ + status x E_2_ | 10 | -26.474 | 76.091 | 3.911 | 0.010 |
| BKA ~ intercept + sex x E_2_ + status x E_2_ | 9 | -27.786 | 76.108 | 3.928 | 0.009 |
| BKA ~ intercept + status + age + sex x E_2_ | 9 | -27.795 | 76.125 | 3.945 | 0.009 |
| BKA ~ intercept + sex | 5 | -32.719 | 76.238 | 4.058 | 0.009 |
| BKA ~ intercept + sex x E_2_ | 7 | -30.390 | 76.314 | 4.135 | 0.009 |
| BKA ~ intercept + status + weight + age | 7 | -30.406 | 76.347 | 4.167 | 0.008 |
| BKA ~ intercept + age + sex x status + sex x E_2_ | 10 | -26.605 | 76.353 | 4.173 | 0.008 |
| BKA ~ intercept + age | 5 | -32.778 | 76.355 | 4.175 | 0.008 |
| BKA ~ intercept + weight + status x E_2_ | 8 | -29.299 | 76.598 | 4.418 | 0.007 |
| BKA ~ intercept + sex + weight | 6 | -31.802 | 76.739 | 4.559 | 0.007 |
| BKA ~ intercept + sex + E_2_ + age | 7 | -30.608 | 76.750 | 4.570 | 0.007 |
| BKA ~ intercept + age + sex x status + status x E_2_ | 10 | -26.809 | 76.760 | 4.580 | 0.007 |
| BKA ~ intercept + sex + weight + status x E_2_ | 9 | -28.140 | 76.815 | 4.635 | 0.007 |
| BKA ~ intercept + weight + sex x E_2_ | 8 | -29.484 | 76.968 | 4.788 | 0.006 |
| BKA ~ intercept + age + status x E_2_ | 8 | -29.513 | 77.027 | 4.847 | 0.006 |
| BKA ~ intercept + sex + status + weight + age | 8 | -29.522 | 77.043 | 4.863 | 0.006 |
| BKA ~ intercept + E_2_ + weight + age + sex x status | 10 | -27.035 | 77.214 | 5.034 | 0.005 |
| BKA ~ intercept + sex + age + status x E_2_ | 9 | -28.367 | 77.270 | 5.090 | 0.005 |
| BKA ~ intercept + sex + status + E_2_ + weight + age | 9 | -28.407 | 77.350 | 5.170 | 0.005 |
| BKA ~ intercept + status + E_2_ + weight + age | 8 | -29.717 | 77.435 | 5.255 | 0.005 |
| BKA ~ intercept + E_2_ + weight + age | 7 | -30.985 | 77.504 | 5.324 | 0.005 |
| BKA ~ intercept + weight + age | 6 | -32.199 | 77.534 | 5.354 | 0.005 |
| BKA ~ intercept + age + sex x E_2_ | 8 | -29.957 | 77.915 | 5.735 | 0.004 |
| BKA ~ intercept + weight + sex x status + sex x E_2_ + status x E_2_ | 11 | -26.071 | 77.968 | 5.788 | 0.004 |
| BKA ~ intercept + weight + age + sex x status + sex x E_2_ | 11 | -26.141 | 78.109 | 5.929 | 0.003 |
| BKA ~ intercept + sex + age | 6 | -32.510 | 78.155 | 5.975 | 0.003 |
| BKA ~ intercept + status + weight + age + sex x E_2_ | 10 | -27.509 | 78.161 | 5.981 | 0.003 |
| BKA ~ intercept + sex + E_2_ + weight + age | 8 | -30.102 | 78.204 | 6.024 | 0.003 |
| BKA ~ intercept + weight + age + sex x status + status x E_2_ | 11 | -26.192 | 78.211 | 6.031 | 0.003 |
| BKA ~ intercept + weight + sex x E_2_ + status x E_2_ | 10 | -27.581 | 78.305 | 6.125 | 0.003 |
| BKA ~ intercept + sex x status x E_2_ | 11 | -26.369 | 78.564 | 6.384 | 0.003 |
| BKA ~ intercept + age + sex x E_2_ + status x E_2_ | 10 | -27.785 | 78.713 | 6.534 | 0.003 |
| BKA ~ intercept + age + sex x status + sex x E_2_ + status x E_2_ | 11 | -26.474 | 78.774 | 6.594 | 0.002 |
| BKA ~ intercept + sex + weight + age | 7 | -31.792 | 79.118 | 6.938 | 0.002 |
| BKA ~ intercept + weight + age + status x E_2_ | 9 | -29.296 | 79.128 | 6.948 | 0.002 |
| BKA ~ intercept + sex + weight + age + status x E_2_ | 10 | -28.010 | 79.163 | 6.983 | 0.002 |
| BKA ~ intercept + weight + age + sex x E_2_ | 9 | -29.463 | 79.462 | 7.282 | 0.002 |
| BKA ~ intercept + weight + age + sex x status + sex x E2 + status x E_2_ | 12 | -25.935 | 80.459 | 8.279 | 0.001 |
| BKA ~ intercept + weight + sex x status x E_2_ | 12 | -26.014 | 80.616 | 8.436 | 0.001 |
| BKA ~ intercept + weight + age + sex x E_2_ + status x E2 | 11 | -27.491 | 80.808 | 8.628 | 0.001 |
| BKA ~ intercept + age + sex x status x E_2_ | 12 | -26.369 | 81.326 | 9.146 | 0.001 |
| BKA ~ intercept + weight + age + sex x status x E_2_ | 13 | -25.898 | 83.229 | 11.050 | 0.000 |

**Supplemental Table 9.** Model selection (AICc) table for HCA with E_2_ (*n* = 78 observations). Italicized terms indicate the top models (ΔAICc < 2). Initial parameters include E_2_ x sex x status, age and weight.

| **fixed effects** | ***df*** | **log likelihood** | **AICc** | **ΔAICc** | **weight** |
| --- | --- | --- | --- | --- | --- |
| *HCA ~ intercept* | *4* | *-400.615* | *809.778* | *0.000* | *0.139* |
| *HCA ~ intercept + age* | *5* | *-399.928* | *810.688* | *0.911* | *0.088* |
| *HCA ~ intercept + status* | *5* | *-400.058* | *810.949* | *1.171* | *0.078* |
| *HCA ~ intercept + sex* | *5* | *-400.226* | *811.285* | *1.507* | *0.066* |
| HCA ~ intercept + E_2_ | 5 | -400.555 | 811.943 | 2.165 | 0.047 |
| HCA ~ intercept + weight | 5 | -400.576 | 811.986 | 2.208 | 0.046 |
| HCA ~ intercept + status + sex | 6 | -399.643 | 812.469 | 2.692 | 0.036 |
| HCA ~ intercept + age + sex | 6 | -399.715 | 812.614 | 2.836 | 0.034 |
| HCA ~ intercept + age + status | 6 | -399.742 | 812.668 | 2.890 | 0.033 |
| HCA ~ intercept + age + weight | 6 | -399.779 | 812.741 | 2.963 | 0.032 |
| HCA ~ intercept + status x sex | 7 | -398.578 | 812.755 | 2.978 | 0.031 |
| HCA ~ intercept + age + E_2_ | 6 | -399.893 | 812.969 | 3.191 | 0.028 |
| HCA ~ intercept + status + E_2_ | 6 | -399.899 | 812.981 | 3.203 | 0.028 |
| HCA ~ intercept + status + weight | 6 | -400.038 | 813.259 | 3.481 | 0.024 |
| HCA ~ intercept + sex + weight | 6 | -400.156 | 813.495 | 3.717 | 0.022 |
| HCA ~ intercept + sex + E_2_ | 6 | -400.219 | 813.622 | 3.844 | 0.020 |
| HCA ~ intercept + E_2_ + weight | 6 | -400.505 | 814.192 | 4.415 | 0.015 |
| HCA ~ intercept + age + status + sex | 7 | -399.471 | 814.541 | 4.763 | 0.013 |
| HCA ~ intercept + age + status + weight | 7 | -399.489 | 814.578 | 4.800 | 0.013 |
| HCA ~ intercept + status + sex + E_2_ | 7 | -399.586 | 814.773 | 4.995 | 0.011 |
| HCA ~ intercept + E_2_ + status x sex | 8 | -398.344 | 814.776 | 4.998 | 0.011 |
| HCA ~ intercept + status + sex + weight | 7 | -399.638 | 814.875 | 5.098 | 0.011 |
| HCA ~ intercept + age + sex + weight | 7 | -399.648 | 814.897 | 5.119 | 0.011 |
| HCA ~ intercept + age + status + E_2_ | 7 | -399.649 | 814.898 | 5.120 | 0.011 |
| HCA ~ intercept + age + status x sex | 8 | -398.413 | 814.912 | 5.134 | 0.011 |
| HCA ~ intercept + status x E_2_ | 7 | -399.697 | 814.994 | 5.216 | 0.010 |
| HCA ~ intercept + age + sex + E_2_ | 7 | -399.710 | 815.020 | 5.243 | 0.010 |
| HCA ~ intercept + age + E_2_ + weight | 7 | -399.765 | 815.129 | 5.352 | 0.010 |
| HCA ~ intercept + weight + status x sex | 8 | -398.567 | 815.220 | 5.443 | 0.009 |
| HCA ~ intercept + sex x E_2_ | 7 | -399.851 | 815.303 | 5.525 | 0.009 |
| HCA ~ intercept + status + E_2_ + weight | 7 | -399.881 | 815.361 | 5.584 | 0.009 |
| HCA ~ intercept + sex + E_2_ + weight | 7 | -400.146 | 815.892 | 6.114 | 0.007 |
| HCA ~ intercept + age + status + sex + weight | 8 | -399.332 | 816.751 | 6.973 | 0.004 |
| HCA ~ intercept + sex + status x E_2_ | 8 | -399.343 | 816.774 | 6.996 | 0.004 |
| HCA ~ intercept + status + sex x E_2_ | 8 | -399.358 | 816.803 | 7.025 | 0.004 |
| HCA ~ intercept + status x sex + sex x E_2_ | 9 | -398.108 | 816.864 | 7.086 | 0.004 |
| HCA ~ intercept + age + status + E_2_ + weight | 8 | -399.428 | 816.943 | 7.166 | 0.004 |
| HCA ~ intercept + age + status + sex + E_2_ | 8 | -399.434 | 816.955 | 7.177 | 0.004 |
| HCA ~ intercept + age + sex x E_2_ | 8 | -399.488 | 817.063 | 7.285 | 0.004 |
| HCA ~ intercept + age + E_2_ + status x sex | 9 | -398.221 | 817.090 | 7.312 | 0.004 |
| HCA ~ intercept + age + status x E_2_ | 8 | -399.518 | 817.124 | 7.346 | 0.004 |
| HCA ~ intercept + status + sex + E_2_ + weight | 8 | -399.580 | 817.248 | 7.470 | 0.003 |
| HCA ~ intercept + status x sex + status x E_2_ | 9 | -398.321 | 817.288 | 7.510 | 0.003 |
| HCA ~ intercept + E_2_ + weight + status x sex | 9 | -398.330 | 817.307 | 7.529 | 0.003 |
| HCA ~ intercept + age + sex + E_2_ + weight | 8 | -399.646 | 817.380 | 7.602 | 0.003 |
| HCA ~ intercept + weight + status x E_2_ | 8 | -399.654 | 817.395 | 7.618 | 0.003 |
| HCA ~ intercept + age + weight + status x sex | 9 | -398.390 | 817.426 | 7.649 | 0.003 |
| HCA ~ intercept + weight + sex x E_2_ | 8 | -399.816 | 817.719 | 7.941 | 0.003 |
| HCA ~ intercept + status x E_2_ + sex x E_2_ | 9 | -399.234 | 819.115 | 9.338 | 0.001 |
| HCA ~ intercept + age + status + sex x E_2_ | 9 | -399.251 | 819.150 | 9.372 | 0.001 |
| HCA ~ intercept + age + sex + status x E_2_ | 9 | -399.258 | 819.163 | 9.385 | 0.001 |
| HCA ~ intercept + age + weight + status x E_2_ | 9 | -399.277 | 819.202 | 9.424 | 0.001 |
| HCA ~ intercept + age + status + sex + E_2_ + weight | 9 | -399.303 | 819.254 | 9.476 | 0.001 |
| HCA ~ intercept + sex + weight + status x E_2_ | 9 | -399.321 | 819.289 | 9.511 | 0.001 |
| HCA ~ intercept + age + status x sex + sex x E_2_ | 10 | -398.027 | 819.337 | 9.559 | 0.001 |
| HCA ~ intercept + status + weight + sex x E_2_ | 9 | -399.345 | 819.337 | 9.560 | 0.001 |
| HCA ~ intercept + weight + status x sex + sex x E_2_ | 10 | -398.101 | 819.485 | 9.708 | 0.001 |
| HCA ~ intercept + age + weight + sex x E_2_ | 9 | -399.422 | 819.491 | 9.714 | 0.001 |
| HCA ~ intercept + status x sex + status x E_2_ + sex x E_2_ | 10 | -398.108 | 819.499 | 9.721 | 0.001 |
| HCA ~ intercept + age + E_2_ + weight + status x sex | 10 | -398.212 | 819.707 | 9.929 | 0.001 |
| HCA ~ intercept + age + status x sex + status x E_2_ | 10 | -398.215 | 819.713 | 9.935 | 0.001 |
| HCA ~ intercept + weight + status x sex + status x E_2_ | 10 | -398.313 | 819.909 | 10.131 | 0.001 |
| HCA ~ intercept + age + sex + weight + status x E_2_ | 10 | -399.119 | 821.521 | 11.743 | 0.000 |
| HCA ~ intercept + age + status + weight + sex x E_2_ | 10 | -399.123 | 821.530 | 11.752 | 0.000 |
| HCA ~ intercept + age + status x E_2_ + sex x E_2_ | 10 | -399.162 | 821.607 | 11.830 | 0.000 |
| HCA ~ intercept + weight + status x E_2_ + sex x E_2_ | 10 | -399.209 | 821.701 | 11.924 | 0.000 |
| HCA ~ intercept + age + weight + status x sex + sex x E_2_ | 11 | -398.018 | 822.035 | 12.258 | 0.000 |
| HCA ~ intercept + age + status x sex + status x E_2_ + sex x E_2_ | 11 | -398.021 | 822.043 | 12.265 | 0.000 |
| HCA ~ intercept + status x sex x E_2_ | 11 | -398.083 | 822.167 | 12.389 | 0.000 |
| HCA ~ intercept + weight + status x sex + status x E_2_ + sex x E_2_ | 11 | -398.099 | 822.199 | 12.421 | 0.000 |
| HCA ~ intercept + age + weight + status x sex + status x E_2_ | 11 | -398.203 | 822.405 | 12.628 | 0.000 |
| HCA ~ intercept + age + weight + status x E_2_ + sex x E_2_ | 11 | -399.027 | 824.053 | 14.276 | 0.000 |
| HCA ~ intercept + age + status x sex x E_2_ | 12 | -397.997 | 824.794 | 15.016 | 0.000 |
| HCA ~ intercept + age + weight + status x sex + status x E_2_ + sex x E_2_ | 12 | -398.014 | 824.828 | 15.051 | 0.000 |
| HCA ~ intercept + weight + status x sex x E_2_ | 12 | -398.071 | 824.942 | 15.164 | 0.000 |
| HCA ~ intercept + age + weight + status x sex x E_2_ | 13 | -397.993 | 827.673 | 17.896 | 0.000 |
